# Protein Isolate from *Parkia biglobosa* Seeds Improves Dyslipidaemia and Cardiac Oxidative Stress in Streptozotocin-Induced Diabetic Rats

**DOI:** 10.3390/antiox8100481

**Published:** 2019-10-12

**Authors:** Bolajoko Idiat Ogunyinka, Babatunji Emmanuel Oyinloye, Foluso Oluwagbemiga Osunsanmi, Unathi Kolanisi, Andrew Rowland Opoku, Abidemi Paul Kappo

**Affiliations:** 1 Department of Consumer Sciences, Faculty of Science and Agriculture, University of Zululand, KwaDlangezwa 3886, South Africa; bolajokotimi@gmail.com (B.I.O.); kolanisiu@unizulu.ac.za (U.K.); 2 Biotechnology and Structural Biochemistry (BSB) Group, Department of Biochemistry and Microbiology, Faculty of Science and Agriculture, University of Zululand, KwaDlangezwa 3886, South Africa; OpokuA@unizulu.ac.za (A.R.O.); KappoA@unizulu.ac.za (A.P.K.); 3 Department of Biochemistry, College of Sciences, Afe Babalola University, PMB 5454, Ado-Ekiti 360001, Nigeria; 4 Department of Agriculture, University of Zululand, KwaDlangezwa 3886, South Africa; alafin21@yahoo.com

**Keywords:** cardioprotective, hypoglycaemia, hypolipidaemia, *Parkia biglobosa*, oxidative stress

## Abstract

Reports from previous studies now provide evidence that dyslipidaemia and oxidative stress play crucial roles in the pathogenesis and progression of diabetes and its related complications. This research is aimed to investigate the potential effects of protein isolate from *Parkia biglobosa* seeds (PBPI) in streptozotocin (STZ)-induced diabetic rats by measuring blood glucose levels, changes in lipid metabolism and biomarkers of oxidative stress. Diabetic rats were treated orally with graded doses of PBPI, 200 mg/kg bw and 400 mg/kg bw, and 5 U/kg, intraperitoneal (i.p.) of insulin once daily for 28 days with the fasting blood glucose (FBG) monitored weekly. The effect of PBPI on the serum lipid profile was measured while the extent of lipid peroxidation (LPO), as well as antioxidant parameters (superoxide dismutase; SOD, catalase; CAT, glutathione-S-transferase; GST and total glutathione; total GSH), was determined in the cardiac homogenates of diabetic rats. At the tested doses, treatment with PBPI was significantly effective in lowering FBG, serum triglyceride, cholesterol, low-density lipoprotein cholesterol (LDL-c) and very low-density lipoprotein cholesterol (VLDL-c), while concurrently increasing high-density lipoprotein cholesterol (HDL-c). PBPI also significantly decreased the elevations witnessed in LPO levels and restored the biomarkers of oxidative stress in the cardiac homogenate of experimental rats. The results from this study demonstrate that PBPI could improve dyslipidaemia and cardiac oxidative stress in the experimental diabetic animal model possibly by reducing and effectively scavenging reactive oxygen species (ROS) production as well as by increasing antioxidant capacity in combating oxidative stress. Therefore, it can be concluded that PBPI could be explored in the development of a potent cardioprotective supplement or adjuvant therapy towards the management of diabetes and its related complications.

## 1. Introduction 

Diabetes mellitus is a group of metabolic disorders characterised by a defect in insulin action, secretion or both, resulting in a disturbance of carbohydrate, lipid and protein metabolism [1]. Chronic hyperglycaemia, the hallmark of diabetes, is associated with long-term complications. Among the various complications associated with diabetes mellitus (DM), cardiovascular complications specifically, hypertension, coronary heart disease as well as diabetic cardiomyopathy (DCM) are the principal causes of morbidity and mortality [2]. Hyperglycaemia-induced free radical generation in diabetes has been linked to glucose oxidation, glycation of proteins and the degradation of glycated proteins. On the one hand, these factors have been suggested to contribute to the decline in the endogenous antioxidant status. A shift in this critical balance leads to oxidative stress, which in turn leads to tissue damage, lipid peroxidation and insulin resistance [3,4,5]. 

Diabetic patients are more prone to the risk of cardiovascular diseases compared to non-diabetic patients. About 80% of mortality in diabetics is attributed to cardiovascular diseases [6]. Various alterations and abnormalities in the serum lipid profile have been reported to be rampant in diabetes mellitus due to the fact that insulin resistance or insulin deficiency alters key enzymes and pathways in lipid metabolism [7]. On average, it has been established that there is a general decrease in the high-density lipoprotein cholesterol (HDL-c) level associated with a corresponding increase in triglyceride, cholesterol and low-density lipoprotein cholesterol (LDL-c) as well as very low-density lipoprotein cholesterol (VLDL-c) levels in diabetes mellitus [8]. This alteration in the serum lipid profile increases the risk of arteriosclerosis, the onset of coronary heart disease (CHD) [9]. 

Despite newer and effective therapeutic approaches in the treatment and management of diabetes, the prevalence of diabetes mellitus and its related complications continues to increase unabatedly [10]. Most of the current antidiabetic drugs in use have exhibited undesirable side effects and limited efficacy. Hence, there is a need to exploit medicinal plants with antidiabetic potentials, which are believed to be less toxic and have limited or transient side effects when compared to the current antidiabetic drugs available [11,12]. Some medicinal plants with antidiabetic properties have previously been reported in the literature [13,14,15,16]. *Parkia biglobosa,* commonly known as “African locust beans”, which belongs to the family of Mimosaceace, is one of such medicinal plant. It is widely distributed in the Guinea and Sudan Savannah [17]. 

The fermented seeds of *P. biglobosa* are used as conventional food seasoning in Nigeria and other West African countries [18]. More so, the use of *P. biglobosa* in traditional medicine is gaining popularity—traditional healers in Senegal and in the South West of Nigeria use it for the treatment of diabetes mellitus [19,20]. It is also used in Northern Nigeria for the treatment of diarrhoea in infants [21]. Phytochemical evaluation of *P. biglobosa* has shown that it contains alkaloids, cardiac glycosides, high amino acids and protein content [10,18]. The antimicrobial activities of the leaf and stem bark, as well as the antidiabetic and antihyperlipidaemic effect of the methanol seed extract of *P. biglobosa*, have also been previously reported [10,22,23]. However, to the best of our knowledge, there is very little or no information on the hypolipidaemic and cardioprotective potential of protein isolate from *P. biglobosa* seeds (PBPI). With this background in mind, this study was designed to evaluate the hypolipidaemic, hypoglycaemic and cardioprotective potentials of the protein isolate from *Parkia biglobosa* seeds in streptozotocin-induced diabetic rats.

## 2. Materials and Methods

### 2.1. Chemicals 

All the chemicals used in this study were of analytical grade. Streptozotocin (STZ; Sigma-Aldrich Co., St Louis, MO, USA). All other chemicals and kits were obtained from ScienCell Research Laboratories (Carlsbad, CA, USA) and Merck (Modderfontein, South Africa). 

### 2.2. Collection of Plant Materials

Prior to the importation of *Parkia biglobosa* seeds into South Africa, importation permit (P0060156) was obtained from the Department of Agriculture, Forestry and Fisheries (DAFF; Pretoria, Republic of South Africa). Thereafter, the raw and fermented seeds of *Parkia biglobosa* used in this study were purchased from a local market in Ijebu-Ode, Ogun State, Nigeria. Identification and authentication of the seeds to obtain a voucher number (B07) was done by the Chief Botanist in the Department of Botany, University of Zululand. 

### 2.3. Preparation of the Protein Isolate 

The method described by Ogunyinka and colleagues was adopted in the extraction of the protein isolate [24]. Briefly, fermented seeds of *Parkia biglobosa* were air-dried for several days and then pulverised into fine powder using an electric blender. The powdered seed sample (1 kg) was defatted with 2000 mL of n-hexane to obtain the defatted extract, which was later air-dried and then extracted (1:10 *w/v*) further with butanol in order to remove possible antinutrients present in the sample. The defatted sample was thereafter dissolved in distilled water (pH 10) overnight. The sample was filtered using Whatman No. 1 filter paper. The filtrate was afterwards adjusted to pH 5 and was centrifuged at 7650× *g* for 15 minutes at 4 °C. The supernatant was discarded while the pellet containing the protein isolate was retained and freeze-dried. The lyophilised sample yielded a brown extract.

### 2.4. STZ-Induced Experimental Diabetic Rats and Experimental Design

Healthy male Sprague-Dawley rats (body weight: 250–290 g) obtained from the Departmental animal house (Department of Biochemistry and Microbiology, University of Zululand) were used in this study. These animals were kept in a standard room under a controlled temperature of 20 °C–24 °C and a 12 h light/12 h dark cycle, with access to food and water ad libitum. All experiments complied with the Guidelines on Ethical Standards for the investigation in animals. The experimental protocol was approved by the University of Zululand Research Ethics Committee (UZREC 171110-030-RA level 02 Dept. 2014/74). The rats were divided into seven groups of 10 rats each as follows. Group 1 served as the control and was given citrate buffer only, Group 2 (PI 200) and Group 3 (PI 400) were non-diabetic rats treated with PBPI (200 mg/kg body weight and 400 mg/kg body weight, respectively) and given citrate buffer only. STZ-induced diabetic rats were divided in four groups (Groups 4–7). Group 4 (STZ only) served as the diabetic control group. Group 5 (STZ I) served as the positive control and was given insulin (5 U/kg, intraperitoneal; i.p.) while Group 6 (STZ PI 200) and Group 7 (STZ PI 400) were diabetic animals that received PBPI (200 mg/kg body weight and 400 mg/kg body weight, respectively). Treatments were given by oral gavage for 28 days. 

### 2.5. Induction of Diabetes in Experimental Animals 

The experimental animals were made to fast overnight prior to diabetes induction [24]. Freshly prepared streptozotocin (STZ; Sigma-Aldrich Co.) in an ice-cold citrate buffer, pH 4.5, was administered intraperitoneally at 60 mg/kg body weight [24]. After 72 hours of streptozotocin administration, blood glucose levels were estimated using a glucometer and rats with a blood glucose ranging between 250–300 mg/dL were considered diabetic and used for the experiments. On the fourth day, treatment started orally by gavage as single daily treatments for 28 days. At the end of the treatment period, the rats were fasted for 16 hours. The animals were then euthanised through cervical dislocation. Blood samples were collected by cardiac puncture. The blood was centrifuged at 2000 × *g* for 2 min at 4 °C to obtain the serum, which was stored at −80 °C until required for biochemical analyses. Subsequently, the visceral organ (hearts) was harvested, blotted dry, weighed, washed immediately with normal saline and then homogenised in 56 mM Tris-HCl buffer (pH 7.4) containing 1.15% KCl. The homogenates were centrifuged at 10,000× *g* for 15 minutes at 4 °C and the resulting supernatant was used for the lipid peroxidation and antioxidant assays.

### 2.6. Determination of Lipid Profile in Serum

Total cholesterol (TC), total triglyceride (TG) and high-density lipoprotein cholesterol (HLD-c) were estimated using a commercial kit (Roche Diagnostics, Mannheim, Germany) based on manufacturer procedures. The very low-density lipoprotein cholesterol (VLDL-c) and low-density lipoprotein cholesterol (LDL-c) were calculated using Friedewald’s equation [25] as follows: 

VLDL-c = TG/5; 

LDL-c = TC – HDL-c – VLD-c.

### 2.7. Measurements of Antioxidants and Malondialdehyde Content

Total glutathione (total GSH) as well as the activities superoxide dismutase (SOD), catalase (CAT) and glutathione-S-transferases (GST) were determined in the homogenised tissue using commercial assay kits (ScienCell Research Laboratories, Carlsbad, CA, USA) following the manufacturer’s instructions. The extent of lipid peroxidation in the tissue homogenate was measured following the method described by Varshney and Kale [26]. This was done by measuring the formation of malondialdehyde (MDA), a lipid peroxidation product. 

### 2.8. Statistical Analysis 

Data were expressed as the mean ± standard deviation. Differences between all groups were analysed by one-way ANOVA followed by Duncan’s multiple range test (SPSS13.0, Inc., Chicago, IL, USA). *p* < 0.05 was considered as indicative of significant difference. 

## 3. Results

**Figure 1:**
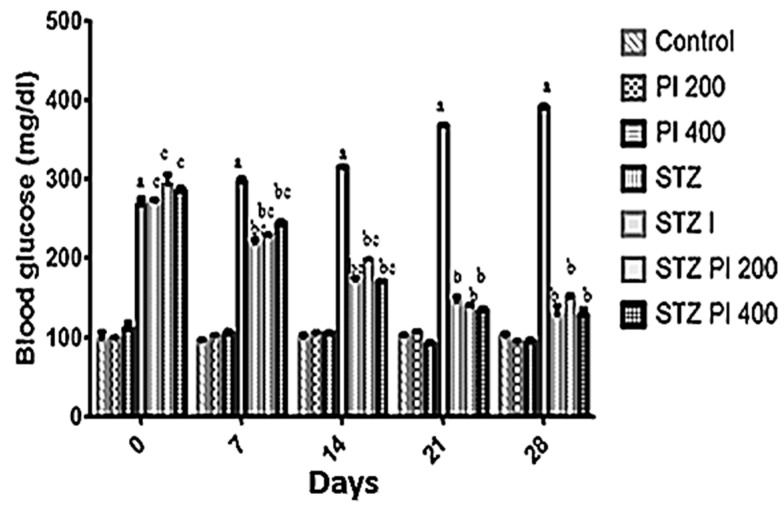
Changes in fasting blood glucose levels on weekly basis in experimental animals treated with protein isolate from *Parkia biglobosa*. Data in the figure above represents mean ± standard deviation, *n* = 10. ^a^—compared with control, ^b^—compared with streptozotocin (STZ) only, ^c^—compared with treated non-diabetic groups. Values are statistically significant at *p* < 0.05.

The effect of protein isolate from *Parkia biglobosa* seeds (PBPI) on blood glucose was examined by monitoring the weekly alterations in the fasting blood glucose (FBG) levels of the experimental rats. The results obtained are presented in Figure 1. It was observed that the administration of STZ (60 mg/kg) to the experimental rats led to significant elevation in FBG levels in all STZ-treated groups when compared with the normal control. The oral administration of PBPI (200 or 400 mg/kg bw) showed a marked attenuation in the elevated FBG levels in the diabetic rats in a dose-dependent manner. In the same vein, there were no significant changes in the FBG levels of the non-diabetic rats treated with PBPI (200 or 400 mg/kg bw) when compared to the normal control. 

**Figure 2:**
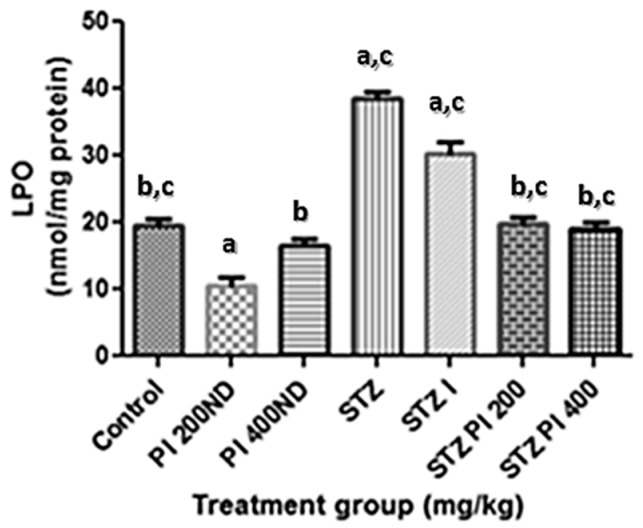
Effects of protein isolate from *Parkia biglobosa* on lipid peroxidation level in experimental animals. Data in the figure above represents mean ± standard deviation, *n* = 10. ^a^—compared with control, ^b^—compared with STZ only, ^c^—compared with treated non-diabetic groups. Values are statistically significant at *p* < 0.05.

**Figure 3:**
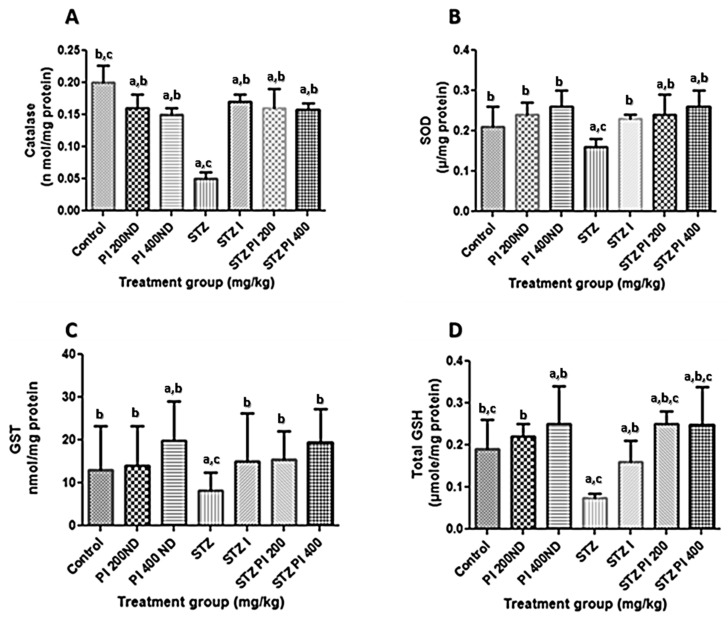
Effects of protein isolate from *Parkia biglobosa* on (**A**) catalase, (**B**) SOD and (**C**) GST activity as well as (**D**) total GSH level in experimental animals. Data in the figure above represents mean ± standard deviation, *n* = 10. ^a^—compared with control, ^b^—compared with STZ only, ^c^—compared with treated non-diabetic groups. Values are statistically significant at *p* < 0.05. SOD: superoxide dismutase; GST: Glutathione S-transferase; GSH: Reduced glutathione.

The effects of PBPI on the serum lipid profile in the experimental animals are presented in Table 1. The diabetic rats showed a significant (*p* < 0.05) increase in the levels of cholesterol, low-density lipoprotein cholesterol (LDL-c) and very low-density lipoprotein cholesterol (VDL-c), and a corresponding decrease in high-density lipoprotein cholesterol (HDL-c) in comparison with the normal control. Treatment with PBPI (200 or 400 mg/kg bw) significantly reversed the observed effect of STZ on the serum lipid profile in the experimental animals in the present study. As shown in Figure 2, the lipid peroxidation (LPO) levels of the STZ-induced diabetic rats were significantly higher than the normal control. The administration of PBPI (200 or 400 mg/kg bw) to the diabetic animals caused the LPO levels to decrease when compared with the normal control. Figure 3 shows the effect of PBPI on catalase, SOD and GST activities as well as its effect on the total glutathione levels of the experimental rats. The diabetic rats showed significantly lower catalase, SOD and GST activities in comparison with the control. The administration of PBPI (200 or 400 mg/kg bw) or insulin significantly restored catalase, SOD and GST activities in comparison with the untreated diabetic rats.

In the same vein, upon treatment with PBPI (200 or 400 mg/kg bw) or insulin, there was an appreciable increase in the total glutathione level when compared with the diabetic rats. Added to this, it is also worth knowing that there was a significant increase in the total glutathione level in the non-diabetic rats treated with PBPI in comparison to the normal control. 

## 4. Discussion

The contributions of various predisposing risk factors, such as hyperglycaemia, hyperlipidaemia and oxidative stress as well as abnormal platelet aggregation, decrease in fibrinolytic activity and hypertension in diabetes mellitus (an endocrine disorder), have been reported in the literature. These risk factors have also been implicated in coronary heart diseases, one of the leading causes of death in diabetes mellitus patients [27,28]. Streptozotocin, a widely accepted diabetogenic agent, has the ability to selectively destroy pancreatic insulin-secreting β-cells, resulting in poor glucose utilisation [29]. Diabetic states are characterised by polyphagia, polyuria, polydipsia and severe weight loss [30]. Many plant products have been reported to possess medicinal properties, such as antidiabetic, antioxidant, hypolipidaemic, hypocholesterolaemic and hypoglycaemic properties [30,31,32,33]. The antidiabetic properties of fermented *Parkia biglobosa* have been demonstrated on alloxan-induced diabetic rats [10]. 

In this study, it was also observed that the continuous administration of protein isolate from *Parkia biglobosa* (PBPI) for 28 days significantly reversed the hyperglycaemic state in STZ-induced diabetic rats. This complemented the hypoglycaemic properties of fermented *Parkia biglobosa* (jacq), as previously reported [10]. The observed effect was in a dose-dependent manner, which was comparable to the standard drug (insulin, 5 U/kg). Whether PBPI increases insulin levels in these animals is a question we intend to understand in future studies. This we propose to do by exploring the effect of PBPI on phosphatidylinositol 3-kinase/protein kinase B (PI3K/AKT) signalling pathways in other to elucidate the mechanism(s) by which PBPI protects cardiac mitochondrial morphology in diabetic conditions.

Uncontrolled hyperglycaemia and cardiac lipid accumulation have been implicated in a variety of pathological pathways connected to the development of cardiac abnormalities. Hyperglycaemia and cardiac lipid accumulation are believed to play a pivotal role in the pathogenesis of metabolic, structural and functional abnormalities observed in the hearts of diabetics [34]. Diabetes mellitus patients have an increased risk of premature atherosclerosis, coronary insufficiency and myocardial infarction due to an alteration in the serum lipid profile [35]. Thus, the observed effect of the PBPI in decreasing the serum lipid and lipoproteins such as cholesterol, triglyceride, VLDL-c and LDL-c, and increasing HDL-c (Table 1) could be beneficial in the prevention of cardiovascular diseases [6]. Although the precise hypolipidaemic mode of action of the PBPI is yet unknown, it could be attributed to the synergetic stimulation of insulin secretion or action by the bioactive constituents of PBPI. PBPI has been acknowledged and believed to possess various promising insulin-like proteins with insulin-releasing activity in our previous study [36]. Insulin has been reported to possess hypolipidaemic effects in STZ-induced diabetic rats [37]. In this study, PBPI is believed to be directly or indirectly involved in lipid absorption and metabolism and this may account for the observed improvement of diabetic dyslipidaemia [38]. 

Hyperglycaemia in diabetes mellitus induces the generation of reactive oxygen species (ROS), which causes cellular damage by oxidising nucleic acid, protein and membrane lipids [37]. Uncontrolled ROS impairs the activities of endogenous antioxidant defense systems, such as SOD, catalase and glutathione reductase, which are known to counterbalance the toxic effect of free radicals [38]. The general decline in the antioxidant status in this study may be a result of the overwhelming effect of the ROS generated due to STZ exposure. Our result is in agreement with previous reports that natural products possess an antioxidative capacity that can mitigate STZ-induced toxicity in rats [39]. It is believed that one of the antidiabetic mechanisms of action of PBPI could be explained by its ability to enhance endogenous enzymatic and non-enzymatic antioxidant production and possibly to reduce and effectively scavenge mitochondrial ROS generation. However, further studies are required to firmly establish these mechanisms of action.

Added to this, the induction of diabetes with STZ resulted in the elevation of lipid peroxidation products. The extent of lipid peroxidation plays an important role in the measurement of the toxicity of many xenobiotics and also serves as one of the most important manifestations of oxidative stress and damage [40,41,42]. An increased LPO level in heart homogenates in this study was an indication of damage and dysfunction in the organ. The significant decrease in the extent of LPO and the marked restoration in cardiac endogenous enzymatic and non-enzymatic antioxidants in this study is an indication that PBPI possesses cardioprotective potential. From our results, we propose that PBPI may improve cardiac function in diabetes mellitus via its cardiac antioxidative roles and may also be indirectly involved in the improvement of glucose and lipid metabolism.

## 5. Conclusions

In conclusion, the present study reveals that PBPI showed hypoglycaemic, hypolipidaemia and antioxidant properties in the STZ-induced diabetic animal model. Therefore, PBPI could serve as a potential cardioprotective supplement or adjuvant therapy in the treatment and management of diabetes mellitus. However, further studies are required to elucidate the exact mechanisms of action.

## Figures and Tables

**Table 1:** Effect of protein isolate from *Parkia biglobosa* on serum lipid profile in experimental animals.

Groups	Cholesterol	HDL-c	LDL-c	VDL-c	TG
		mg/dL			
**Control**	94.38 ± 3.40 ^b,^*	44.4 ± 4.50 ^b,^*	15.6 ± 0.01 ^b^	35.1 ± 1.10 ^b,c,^*	121.86 ± 0.12 ^b,c,^*
**PI 200**	82.38 ± 4.53 ^a,b,c,d^	43.4 ± 0.35 ^b,^*	14.7 ± 2.18 ^b^	37.05 ± 4.50 ^b,^*	114.81 ± 8.9 ^a,b,c,^*
**PI 400**	92.17 ± 2.54 ^b,^*	41.6 ± 4.50 ^b,^*	15.6 ± 0.01 ^b^	34.97 ± 2.54 ^b,c,^*	124.69 ± 5.80 ^b,c^
**STZ**	136.11 ± 1.03 ^a,c,d,^*	32.3 ± 2.25 ^a,c,d,^*	32.1 ± 2.25 ^a,d,^*	73.58 ± 2.59 ^a,c,d,^*	267.22 ± 11.27 ^a,c,d,^*
**STZ I**	97.36 ± 4.92 ^b,^*	40.5 ± 2.25 ^b,^*	24.7 ± 2.25	46.83 ± 3.17 ^a,b,d,^*	149.59 ± 8.13 ^a,d,^*
**STZ PI 200**	93.36 ± 1.58 ^b^	42.9 ± 0.01 ^b^	13.4 ± 4.34 ^b^	37.30 ± 1.61 ^b,c^	120.74 ± 1.58 ^c^
**STZ PI 400**	81.9 ± 8.80 ^a,b,c^	53.3 ± 2.25 ^a,b,c^	13.0 ± 2.25 ^b^	15.56 ± 8.16 ^a,b,c^	130.41 ± 8.16 ^a,c^

Data in the table above represents mean ± standard deviation, *n* = 10. ^a^—compared with control, ^b^—compared with STZ only, ^c^—compared with STZ I, ^d^—compared with STZ PI 200, ^*^—compared with STZ PI 400. Values are statistically significant at *p* < 0.05.
